# U8 variants on the brain: a small nucleolar RNA and human disease

**DOI:** 10.1080/15476286.2022.2048563

**Published:** 2022-04-07

**Authors:** Emily J. McFadden, Susan J. Baserga

**Affiliations:** a Department of Molecular Biophysics and Biochemistry, Yale University, New Haven, CT, USA; b Department of Genetics, Yale School of Medicine, New Haven, CT, USA; c Department of Therapeutic Radiology, Yale School of Medicine, New Haven, CT, USA

**Keywords:** U8 snoRNA, ribosomopathy, ribosome biogenesis, Labrune syndrome, leukoencephalopathy, brain calcifications and cysts, *SNORD118*, nucleolus

## Abstract

Small nucleolar RNAs (snoRNAs) are non-coding RNAs vital for ribosomal RNA (rRNA) maturation. The U8 snoRNA, encoded by the *SNORD118* gene in humans, is an atypical C/D box snoRNA as it promotes rRNA cleavage rather than 2′–O–methylation and is unique to vertebrates. The U8 snoRNA is critical for cleavage events that produce the mature 5.8S and 28S rRNAs of the large ribosomal subunit. Unexpectedly, single nucleotide polymorphisms (SNPs) in the *SNORD118* gene were recently found causal to the neurodegenerative disease leukoencephalopathy, brain calcifications, and cysts (LCC; aka Labrune syndrome), but its molecular pathogenesis is unclear. Here, we will review current knowledge on the function of the U8 snoRNA in ribosome biogenesis, and connect it to the preservation of brain function in humans as well as to its dysregulation in inherited white matter disease.

## Introduction

Ribosomes are essential cellular machinery that translate messenger RNA (mRNA) into proteins. Making ribosomes, known as ribosome biogenesis (RB), is expectedly a highly coordinated and energy-demanding process. In eukaryotes, RB accounts for over 60% of energy used in the cell [1] and takes places in the nucleolus (or nucleoli), a membrane-less organelle within the nucleus [2,3]. Deficits in RB results in a class of human diseases known as ribosomopathies [4] and may affect RB steps such as: rDNA transcription by RNA polymerase I (RNAPI), serial processing of the primary 47S rRNA into the mature 28S, 5.8S, and 18S subunits, and exportation to the cytoplasm for assembly (with the 5S rRNA) into the mature 40S and 60S subunits [5–7].

The cascade of primary 47S rRNA processing into the mature ribosomal subunits is multi–step and produces transient pre-rRNA intermediates [8]. These intermediates are shuttled through the refinement process with the help of multiple protein and small nucleolar RNA (snoRNA) co-factors that cleave, chemically modify, and fold the rRNA. SnoRNAs are between 60 and 300 nucleotides (n.t.) in length and are divided into two classes based on conserved nucleotides: H/ACA box and C/D box [9]. Generally speaking, H/ACA box and C/D box snoRNAs direct the pseudouridylation and 2′–O–methylation of rRNA nucleotides, respectively [10–12].

The 140 n.t. U8 snoRNA, transcribed from the *SNORD118* gene in humans, is classified as a C/D box snoRNA as it contains the conserved C box (RUGAUGA, R = purine) and D box (CUGA) motifs, but U8 is unique as it does not participate in 2′–O–methylation of rRNA. Instead, U8 is required for cleavage of immature rRNA to provide 28S and 5.8S rRNAs that comprise the mature 60S subunit. Interestingly, the U8 snoRNA is not found in yeast, as are many other RB trans-acting factors, but is limited to vertebrates. Recently, Jenkinson et al. determined that mutations in the *SNORD118* gene/U8 snoRNA transcript cause the rare inherited white matter disorder (IWMD) known as leukoencephalopathy with brain calcifications and cysts (LCC; aka Labrune Syndrome) [13].

In this work, we will review the early work identifying and defining the role of the U8 snoRNA as well as more recent work that has shed new light onto the U8 snoRNA, pre-U8 snoRNA processing, and its role in LCC. Overall, we will raise critical questions that remain, including: What is the function of the U8 snoRNA? What are its protein and RNA binding partners necessary for function? How do mutations in the U8 snoRNA lead to LCC pathology? Why is the U8 snoRNA not conserved in non-vertebrates? With this review, we hope to bring together the works of geneticists, biochemists, and clinicians to provide a comprehensive picture of what is known about the U8 snoRNA.

## The U8 snoRNA is a C/D box snoRNA involved in maturation of the 5.8S and 28S rRNA

**Figure 1.**
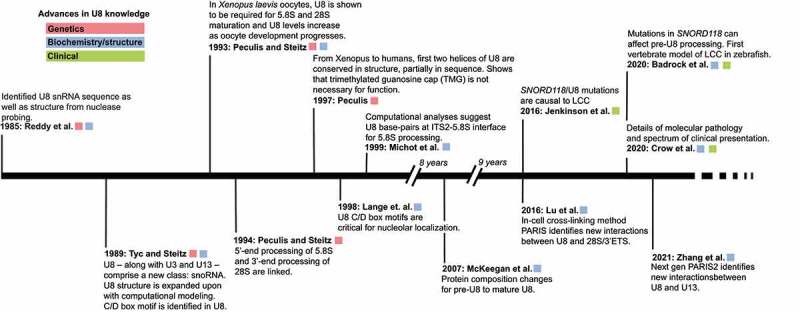
Timeline overview of SNORD118/U8 snoRNA discovery, annotation, and implication in LCC.

The snoRNA U8 was first identified in 1985 by Reddy et al. in rat Novikoff hepatoma cells as a small nuclear RNA (snRNA) and initially called 5.4S RNA as it migrated between 5S RNA and 5.8S RNA on a denaturing acrylamide gel [14] (Fig. 1). Using a trimethylguanosine (TMG) antibody and nuclear/nucleolar fractionation, Reddy et al. showed that U8 has a TMG cap and is found in the nucleolus but not the nucleus. Sequencing gels and nuclease probing gels provided the first sequence and secondary structure model of U8, respectively. Kato and Harada had previously identified a mouse 5.4S RNA [15], to which there was 90% sequence conservation [14]. The RNA was later renamed U8.

Four years later, Tyc and Steitz defined the human U8 snoRNA sequence and structure in HeLa cells [16]. Tyc and Steitz immunoprecipitated U8–along with U3 and U13–using a monoclonal antibody directed against the nucleolar protein, fibrillarin [17,18]. These three RNAs were designated a new class of small RNA, small nucleolar RNA (snoRNA). Tyc and Steitz further classified U8 as a C/D box RNA due to the conserved C box (RGAUGA, R = purine) and D box (CUGA) sequences. These boxes serve as recognition sequences for nucleolar-localized fibrillarin [19]. Prior to the discovery of the U8 snoRNA, however, the U3 snoRNA had been the only known nucleolar C/D box-containing snoRNA.

In 1993, Peculis and Steitz demonstrated the first functional role for the U8 snoRNA in pre-60S rRNA processing. They used RNAase H depletion of U8 in *Xenopus laevis* oocytes activated by microinjection of antisense deoxyoligonucleotides (ASOs) and found that RNA processing at both internal transcribed spacers and at the 3′–end of the 28S rRNA coding region were reduced by U8 snoRNA depletion [20]. This resulted in newly identified pre-rRNA intermediates, suggesting that alternate processing pathways are available but typically disfavoured. Follow-up work from Peculis and Steitz (1994) confirmed that 5′–end processing of 5.8S and 3′–end processing of 28S are linked via U8 [21].

The U8 snoRNA is thus one of a small group of C/D box snoRNAs that are required for pre-rRNA cleavage and not for 2′–O–methylation of the pre-rRNA ribose even though it associates with the nucleolar methyltransferase protein fibrillarin: U3, U8, U13, and U22 [12,22,23]. Peculis (1997) completed mutagenesis studies on the conserved 5′–end of U8 and found that disruption of the 5′–most 15 nucleotides of U8 inhibited pre-rRNA processing, but that this region alone was not sufficient for rRNA processing. She did show that this U8 snoRNA:pre-rRNA interaction is important to modulate rRNA:rRNA interaction and folding, and to allow for pre-rRNA processing to proceed. Supporting these results, Srivastava et al. (2010) found that the DEAD box helicase Ddx51 is critical for dissociation of the U8 snoRNA from the pre-rRNA. Expression of a dominant negative Ddx51 (S403L) resulted in persistent U8 association with the pre-rRNA and disruption in pre-ribosome processing in mouse LAP3 cells [24]. This work is consistent with the model Brenda Peculis proposed where a tightly synchronized exchange of base-pairs among rRNA and U8 snoRNA is necessary for proper processing and folding of the rRNA.

Lu et al. recently developed a psoralen-based cross-linking method called Psoralen Analysis of RNA Interactions and Structures (PARIS) that identifies RNA:RNA interactions in the cell [25]. Using PARIS, Lu et al. found that the 5′–end of U8 snoRNA may in fact directly interact with the 3′–end of 28S as opposed to the 5′–end as suggested by prior genetic work [26] and computational and phylogenetic modelling [27]. Follow up work from Zhang et al. using PARIS2–the next iteration of the PARIS method–found a novel U8:U13 interaction, which they suggest may coordinate the maturation of the 18S and 28S rRNA [28]. However, in this work and others [20,21,26,29] depletion of U8 snoRNA reduced mature 28S rRNA levels, but did not lead to reduced levels of mature 18S, as would be expected if this interaction coordinates synthesis and assembly of both subunits [30]. As dimer interactions between full-length, endogenously expressed transcripts–U8 and the U13 snoRNAs or between U8 and the 28S 3′–end–were investigated computationally and not biochemically, future studies would benefit from testing LCC-relevant U8 snoRNA variants in human cells for their ability to interact with the U13 snoRNA and the 28S 3′–end. Additionally, previous work from Peculis et al. found tolerated sequence variation in *Xenopus laevis* U8, which may serve to attenuate rRNA processing [31]; whether these naturally occurring, functional variants are also found in humans and how these sequence variants would tolerate LCC-associated SNPs has yet to be determined.

Because PARIS and PARIS2 are limited to RNA:RNA duplex interactions, we are also missing important information regarding in-cell protein binding partners. The network of in-cell U8 RNA:RNA interactions initiated by Zhang et al. would be further enriched using RNA:protein methods such as RNP-MaP [32] as previous work has shown a dynamic protein interaction network during U8 small nucleolar ribonucleoprotein particle (snoRNP) formation [33]. The mature U8 snoRNP is known to contain multiple proteins including: fibrillarin [20], multiple LSm proteins (Xenopus) [34], 15.5 K/SNU13 [35], NOP56 and NOP58 [35], *Xenopus* X29 (NUDT16 in humans) [36], and Ddx51 [24] all with varying degrees of functional annotation. However, these experiments pulled down proteins of interest under varying salt conditions and then looked for U8 snoRNA, whereas a targeted U8 snoRNA pull-down followed by protein identification has yet to be carried out.

**Figure 2.**
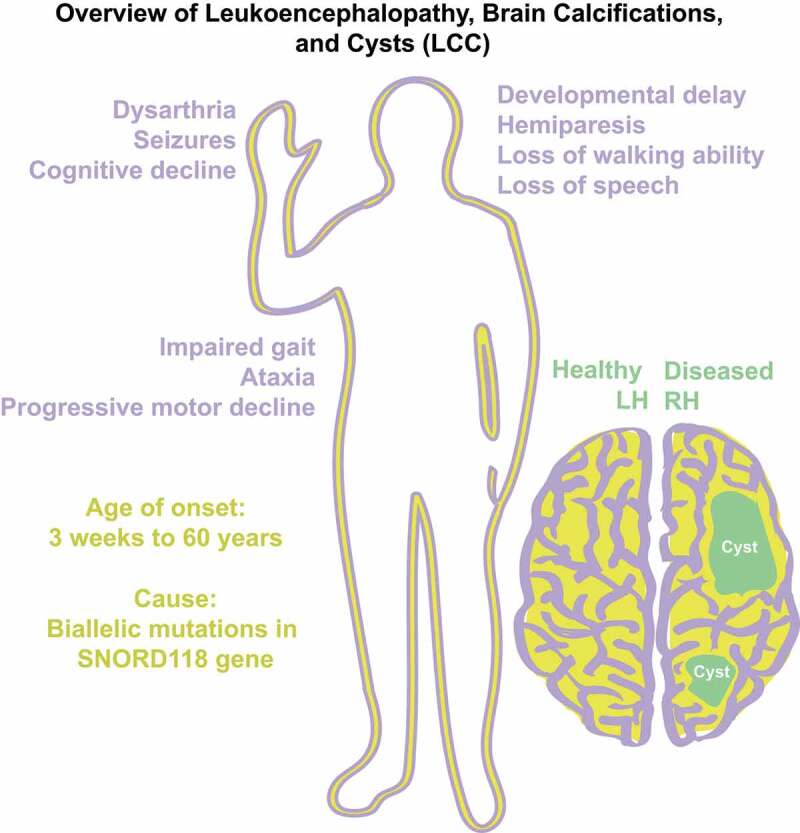
Pathologies of LCC (purple) are mapped alongside genetic information (yellow) and cartoon of brain disease (green).

## SNORD118/U8 snoRNA is mutated in Leukoencephalopathy, Brain calcifications and cysts (LCC; Labrune syndrome)

LCC, also known as Labrune Syndrome, was first described by Labrune et al. in 1996 (Fig. 2) [37]. Initially, Labrune et al. found that LCC only presented in early infancy to adolescence, but was later found in patients as late as 60 years of age [38]. LCC belongs to a class of diseases known as leukodystrophies or inherited white matter disorders (IWMD) [39]. IWMD are genetic disorders primarily affecting central nervous system (CNS) white matter. LCC is exclusively a neurological Mendelian disease with unknown population frequency that follows a degenerative course. Clinically, the disease presents with general decline of cognitive abilities and motor abilities due to advancing growth of cysts in one or both hemispheres of the brain [40]. LCC is distinguishable from Coats disease, another IWMD with similar presentations, as mutations in the conserved telomere maintenance component 1 (CTC1) had been found causal to Coats disease but not LCC [41]. Interestingly, although a rare autosomal recessive disease, there is no known enrichment for consanguinity [42]. The disease was classically diagnosed with histopathological, neuroradiological, and clinical findings and had no known genetic cause or biomarker [37]. Unfortunately, there are no known cures for LCC, only treatments to alleviate symptoms. Some success in ameliorating gait, range of motion and disease progression has been attained through the administration of bevacizumab [43].

Although human U8 snoRNA was identified in 1985, it was only in 2016 where Jenkinson et al. identified biallelic, autosomal recessive mutations in the *SNORD118* gene encoding the U8 snoRNA as a cause of LCC (Fig. 1) [13]. LCC is thus the first human disease that can be ascribed to mutations in a gene encoding a C/D box snoRNA. Jenkinson (2016) sequenced the exomes of 12 LCC patients used linkage and haplotype analysis to identify a single region >1 Mb, located on chromosome 17. Focusing on chromosome 17, they used capture sequencing to find two rare variants in a single 199 base-pair (b.p.) region of DNA that encompassed the gene *SNORD118*. Sanger sequencing confirmed these variants. They extended their approach to 33 families with 40 affected individuals, finding compound heterozygosity for variants in *SNORD118* for each of the LCC-affected individuals. In total, there were 36 rare putative pathogenic variants identified in *SNORD118* with 29 variants occurring in the U8 snoRNA sequence itself.

**Figure 3.**
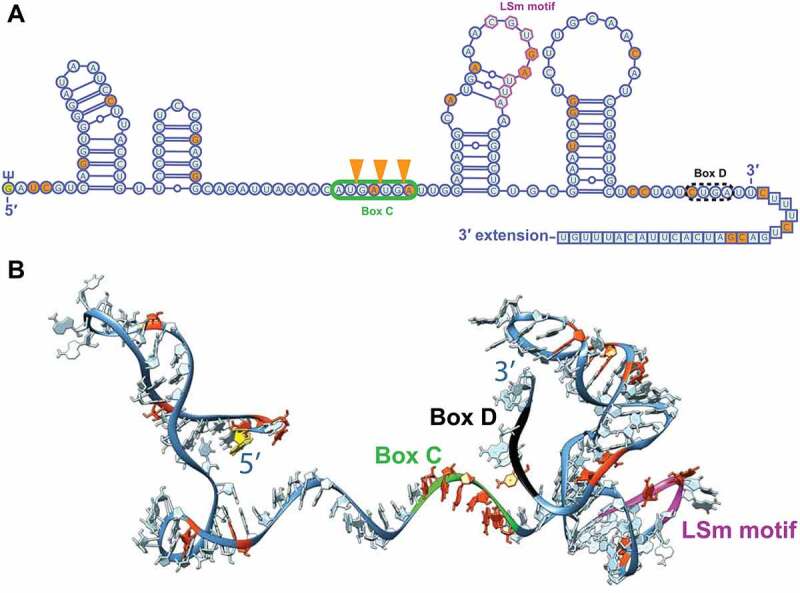
Structure of U8 snoRNA. A) Secondary structure of mature U8 snoRNA. Sequence and structure taken from [21]. Orange circles refer to naturally occurring SNPs found in [13]. Orange triangles refer to nucleobase insertions. Yellow circle is trimethylguanosine cap. Green box and black box refer to C and D box motifs, respectively. Purple hexagons are LSm motif. Boxed nucleotides refer to pre-U8 snoRNA 3′–extension from [42]. Image generated using Varna [44]. B) Three-dimensional computational model of U8 snoRNA. Sequence and secondary structure from [21]. Model generated using RNAComposer [45,46]. Colour scheme corresponds to those mapped onto A.

Functional analysis confirmed that some of the variants present in LCC-affected individuals were deleterious for maintaining either U8 snoRNA levels or function [13]. A variant present in the predicted *SNORD118* promoter sequence reduced reporter-gene expression by 109-fold. Interestingly, 10 of the variants occurred in either the C or D box sequence or in the LSm binding site, conserved sequences known to be critical for protein binding (Fig. 3). Indeed, testing four variants in the U8 snoRNA C box sequence for association with the 15.5 K/SNU13 protein *in vitro* revealed significantly reduced protein binding. Furthermore, four variants in the pre-U8 snoRNA, which contains an extended 3′–end as compared to mature U8 snoRNA, caused reduced 3′–end processing in HeLa cell extracts. Lastly, LCC fibroblasts from four patients demonstrated reduced U8 snoRNA levels by qRT-PCR and were found to grow more slowly, consistent with reduced ribosome biogenesis. Thus, it is likely that the described variants are pathogenic and cause loss-of-function mutations.

Since LCC primarily affects brain development, and the U8 snoRNA has been so far found only in vertebrates, Badrock et al. [42] pursued a vital zebrafish model to test the significance of the putative pathogenic LCC-associated U8 variants identified by Jenkinson et al. (2016), as well as to sort the null/severe from the milder/partial functional mutant alleles. Importantly, they initially demonstrated that the presence of the U8 snoRNA is required for normal organismal development and survival, as zebrafish with a CRISPR/Cas9 disruption in the U8-3 gene locus showed defective brain development with death occurring from 6 to 9 days post fertilization. As expected, the 28S/18S rRNA ratio was reduced and pre-28S rRNAs accumulated. Many of the gross morphological phenotypes conferred by the disrupted U8-3 genes could be ‘rescued’ by the presence of the 3′–extended human pre-U8 snoRNA. This made it possible to test U8 snoRNA and gene LCC variants in the context of a whole organism during brain development for the first time.

A series of LCC variants in human pre-U8 snoRNA were tested for their ability to function when expressed in zebrafish [42]. Interestingly, all mutations that had exhibited reduced binding to the 15.5 K/SNU13 protein *in vitro* [13] failed to complement the disrupted U8-3 gene, and were labelled null. Although we have no experimentally determined pre-U8 snoRNA structure, the investigators found that RNAfold predicted base-pairing of the 5′–end of the U8 snoRNA with a 3′–end extension that would be present in the pre-U8 snoRNA (Fig. 3A). Intriguingly, 29/33 LCC individuals harboured a mutation that would affect this novel intramolecular interaction. In contrast to the mutations that conferred reduced binding to the 15.5 K protein, 3 variants in the proposed 3′–extension of pre-U8 snoRNA all fully rescued the gross morphological defects. These were labelled hypomorphic by default. Missing from rigorous proof that these are partially functional alleles is assessment of U8 snoRNA levels and U8 3′–end processing in the rescued zebrafish. These mutations are outside regions of known prior U8 snoRNA functional importance, e.g. C/D box motifs, but demonstrate that there are many unresolved steps to LCC development, including the maturation of U8 snoRNA itself.

Stabilization of the p53 protein is a hallmark of the nucleolar stress response where an increase in free uL5 and uL18 sequester MDM2, an E3 ubiquitin-protein ligase, resulting in an increase in p53 protein levels [47]. This is thought to be the molecular basis of the craniofacial dysmorphology present in the ribosomopathy, Treacher-Collins syndrome [48]. It has also been modelled in zebrafish and *Xenopus tropicalis* upon depletion and disruption of additional proteins required for RNAPI transcription [49,50], indicating a conserved mechanism. Similarly, U8-3 gene disruption in developing zebrafish indicates an increase in levels of the downstream effectors of p53 [42]. Interestingly, a reporter gene system for p53 downstream effectors in the developing embryo reveal the eye, the central nervous system (CNS) and somites as most fluorescent 24–48 hours post-fertilization [42]. Crossing of a disrupted U8 snoRNA gene zebrafish with those genetically inactivated for p53 partially rescues angiogenic sprouting and ventricular swelling [42], indicating a role for p53 stabilization in some of the phenotypic abnormalities. However, the embryos do remain shorter, and with reduced 28S rRNA levels, as expected for defective ribosome biogenesis.

Shortly thereafter, a companion paper from the same group [51] presented a comprehensive collection detailing the genetic variance and phenotypic presentation of LCC in a patient cohort of 64 LCC patients from 56 families. Remarkably, Crow et al. report that there are no obvious correlations between genetic variance and age and/or severity of disease presentation. This genotype-phenotype disparity remains one of the questions about LCC pathology.

## Conclusions

The history of U8 snoRNA discovery and function stretches back to 1984 [15]. It is one of the few C/D box snoRNAs that is important for pre-rRNA cleavage and not for 2′–O–methylation, and is so far found only in vertebrates. A yeast homolog has not been identified but a putative candidate has been identified in *C. elegans*, CeR-2 [52]. These unique characteristics were brought into focus when, in 2016, mutations in *SNORD118*/U8 were found to cause LCC (aka Labrune syndrome). How exactly mutations in the *SNORD118* gene and resultant U8 snoRNA are related to the formation of calcifications and cysts remains unidentified. Recently, white matter disease similar to LCC has been observed in a new Alopecia Neuro Endocrine disease patient, also a ribosomopathy [53], suggesting at least some common presentation of disease.

While we are not sure why the U8 snoRNA is not found outside of vertebrates, evidence is accumulating that there are significant differences between ribosome biogenesis in human cells and the model organism, *S. cerevisiae*, which has been the workhorse organism for understanding nucleolar function. Recent work from Cao et al. has found a nucleolar microprotein that regulates biogenesis of the large ribosomal subunit (LSU) in mammalian cells but so far has not found genetic or biochemical evidence of this microprotein in yeast [54]. Although ribosome biogenesis is generally a highly conserved process among eukaryotes, unique differences among organisms are being uncovered.

To date, functional analyses of U8 in *Xenopus laevis* oocytes [20,21,26] or model organisms [42] has been limited by its lack of conservation outside of vertebrates. As all of the genetic and biochemical studies regarding U8 snoRNA were completed decades before U8 was implicated in LCC, there is a need to integrate the recently LCC-associated variants with future functional genetic and biochemical studies to better understand the disease pathogenesis. While a zebrafish model is extant [42], a mouse model of LCC to connect the LCC-associated *SNORD118* variants with the signs and symptoms of patient presentation is a much-needed approach to determine the pathophysiology of U8 snoRNA-variants during brain development.
